# Gold from nature’s pantry: a diachronic study of *Rubus chamaemorus* L. (Rosaceae) in swedish gastronomy and economy

**DOI:** 10.1186/s13002-025-00843-8

**Published:** 2025-12-28

**Authors:** Ingvar Svanberg, Annika Karlholm, Sabira Ståhlberg

**Affiliations:** 1 Institute for Russian and Eurasian Studies, Uppsala University, Box 514, Uppsala, SE-751 20 Sweden; 2 Institute for Dialectology, Onomastics and Folklore Research, Department of archive and research, Uppsala, Sweden

**Keywords:** Artisanal food, Folk knowledge, Foraging, Gastronomic ethnobiology, Heritage food, Historical ethnobiology, Sustainable use, Wild food

## Abstract

**Background:**

Cloudberry, *Rubus chamaemorus* L. (Rosaceae), is traditionally harvested as food in northern Scandinavia. It is rich in vitamins C, A and E, and antioxidants. This berry has gained much cultural, economic, nutritional, social and symbolic importance in Sweden during the past century. Cloudberries are an important part of culinary heritage.

**Methodology:**

This qualitative diachronic study analyses historical data available in archives and published collections, fragmentary notes in literature and relevant modern data, using a historical ethnobiological approach.

**Results:**

Cloudberry is the third most important wild berry species gathered for human consumption in Sweden. The berries were earlier preserved in wooden barrels during the long circumpolar winter; now they are usually frozen or made into jam and other processed products and sold throughout the country. Difficult access to growth areas, weather fluctuations, timing of harvest and lack of gatherers make harvesting challenging. Commercial harvesting has gone from being a sideline income source for the northern peasants to engaging imported seasonal workers mainly from Southeast Asia.

**Conclusion:**

This historical overview reveals that local berry picking is decreasing in recent decades. Fluctuations in local weather and lack of foragers influence the availability of cloudberry on the market. In 2025, there were neither enough workers nor berries, and the prices of cloudberry products have risen steeply. The cloudberry, which has enjoyed several decades of popularity both in *haute cuisine* and among ordinary consumers, will perhaps return to a local wild food gathered only for household needs.

## Introduction

Sweden is one of the most developed post-industrial countries of the world with a high degree of urbanisation and reliance on technology. Wild foods are however popular and an important part of the cuisine. Gathering berries is an important aspect of local culture and a contributing factor to the economy; it is historically linked to self-sufficiency. There is considerable interest in Nature’s pantry or wild foods both in Swedish *haute cuisine* and home cooking. Wild foods remain a traditional and essential part of the cuisine especially in the northern and central parts of the country [1, 2].

A wide range of organisms can be harvest in the forest landscape: game (moose, red deer, roe deer, boar, hare), berries and macrofungi play a significant role as food resources. There are currently around 300,000 registered hunters in Sweden. The majority participates in moose hunts during the autumn; most are males; only 10.4 per cent are women [3]. Far more among the 10.7 million inhabitants in Sweden are foraging berries and fungi; in contrast to hunters, they are not compelled to register. According to estimates, 20–30 per cent of the population aged 18–74 collect berries and close to 40 per cent forage mushrooms [1]. Since the mid-1900s, mushroom gathering has increased in popularity and constitutes a popular leisure activity for urbanites [2, 4].

**Fig. 1:**
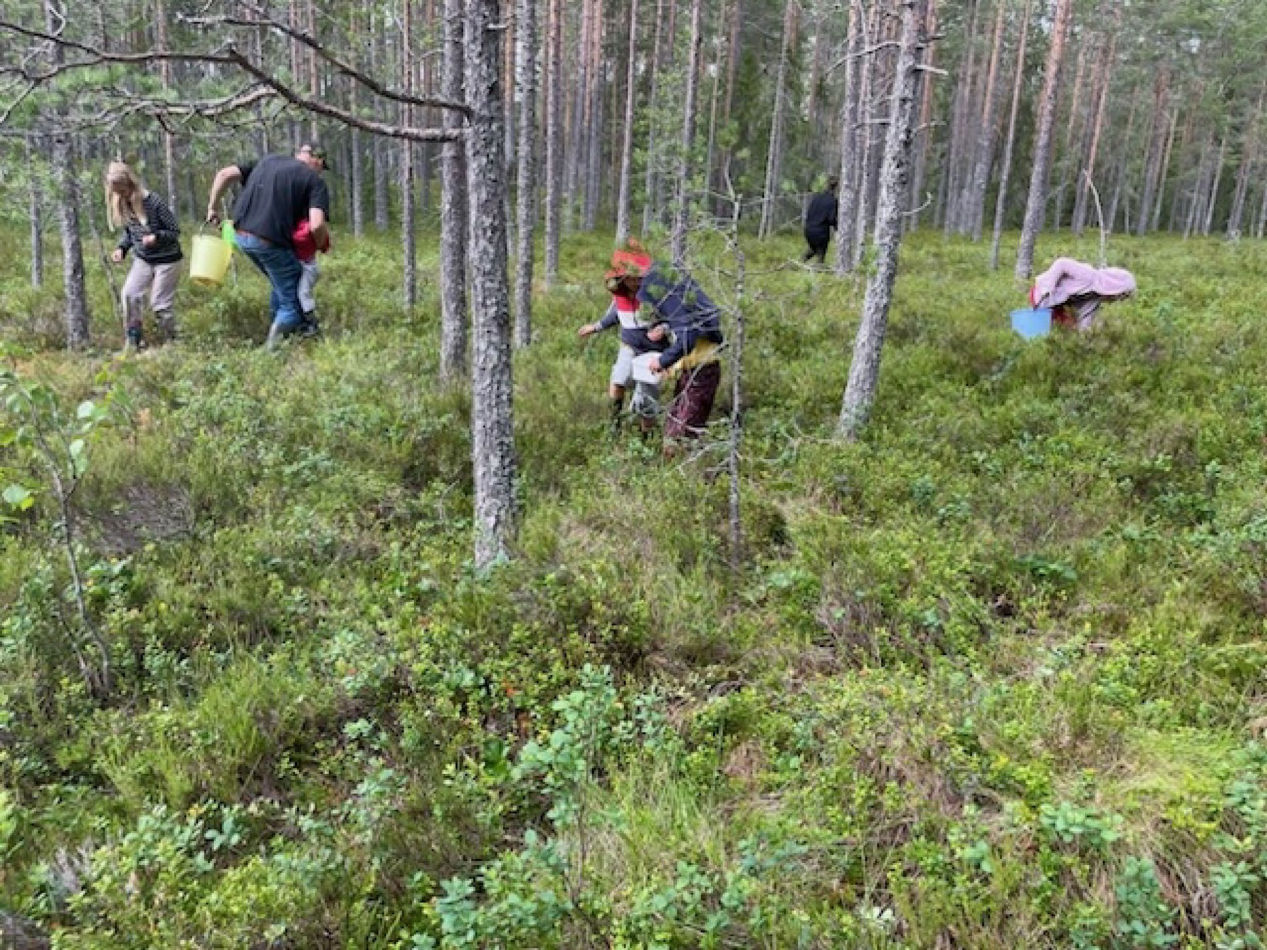
Busy gatherers in a forest in Dalarna. Good cloudberry growth areas are best kept secret (Photo Isak Lidström 2024)

A law regulating public access (Swedish *Allemansrätten*) permits everyone to roam freely on state and private land outside inhabited areas and gather vegetation resources such as berries and mushrooms [5]. Berry gathering for commercial purposes is permitted, as long as it does not cause damage to the area or any inconvenience for the landowners. Foraging is carried out for both private and commercial purposes, and berry gathering is emphasized, along with hunting, as an important traditional activity and value especially for those living in rural areas. Wild berries also contribute to public health through the taxa rich in nutrients ( Fig. 1).

The commercial berry industry creates jobs, but also raises concerns about exploitation of workers, working conditions and sustainability [6].

The most important berries gathered today in Sweden are cowberry, *Vaccinium vitis-idea* L [7]., bilberry, *Vaccinium myrtillus* L [8]., and cloudberry, *Rubus chamaemorus* L [9]. In the island of Gotland in the Baltic Sea, wild-picked dewberries, *Rubus caesius* L., now play an important role as a local commercial product [10]. In the far north, the Arctic bramble, *Rubus arcticus* L., is also locally harvested [11]. Cowberries are economically and traditionally the most important berry collected in Sweden. Bilberries are also in high demand for export [7, 12].

Wild berries are a valuable natural food resource, which however is not exploited to the full in Sweden; despite their abundance, only a small fraction is gathered. The harvest of wild berries for commercial purposes is largely dependent on weather conditions and availability of seasonal workers. The numbers of harvested wild berries vary greatly, but in a normal berry year, over 500,000 tonnes, mainly bilberries (250,000 tonnes), cowberries (150,000 tonnes) and cloudberries (80,000 tonnes) are estimated to be maturing in the forests. In recent decades, on average some 4–6 per cent of these were harvested, but in 2024 the amount fell to only two per cent [13].

Culinary preferences, gathering techniques, preservation, the use of berries, gatherer categories and habits vary widely throughout history in Sweden [7, 10, 14]. Berry picking has been a part of the Swedish rural culture and lifestyle for several centuries, and the popularity of berries has grown from little or no use to daily consumption in homes and regular inclusion into restaurant menus. Traditionally, wild berries were gathered for household use, and the activity was rather local and small-scale [15]. Since the beginning of the nineteenth century, when inexpensive sugar became more widely available globally and also in Sweden, economic interest towards berries and berry products increased. Berry products are presently available widely throughout the country both in specialised shops and supermarkets [9, 16].

The highly colourful, rich in taste and health-supporting cloudberry is one of the most sought-after and prized wild berries harvested in Swedish forests today. “An incredible number of people consider cloudberries to be the most delicious of all our wild berries”, wrote an author already in 1942. Its popularity grew steeply in the second half of the twentieth century [17]. Cloudberry is connected with gold due to its bright yellow colour, and marketed by the food and tourism industries as the ‘Gold of the forest’ (*skogens guld*), ‘Gold of the marshlands’ (*myrarnas guld*), ‘Gold of the mountains’ (*fjällens guld*), ‘Gold of Norrland’ (*Norrlands guld*), ‘Gold of Lapland’ (*Lapplands guld*), ‘Arctic gold’ (*Arktiskt guld*) and similar epithets. Enthusiasts even call it the ‘Grapevine of the North’ (*Nordens vinranka*) and ‘Oranges of the North’ (*Nordens apelsiner*) [18].

Until the 1960 s, the sale of self-collected cloudberries was an important source of income for many rural inhabitants in the northern municipalities. Local foragers continue to eat cloudberries with milk, *filmjölk* (a popular traditional fermented Swedish milk product), or yogurt, in oatmeal or rye porridge, or as a snack on the spot while collecting [19, 20]. Cloudberry has been important food for many indigenous peoples and settlers throughout the northern circumpolar region in Eurasia and North America [21]. Its cultural, economic, and nutritional importance in Sweden is however sparsely highlighted in economic-historical, ethnological and ethnobiological research.

## Aims

The purpose of the present study is to discuss the historical rise of cloudberry from a regional source of nutrition to a widely appreciated modern ingredient and gastronomic delicacy in Sweden. It was probably one of the most important sources of vitamin C in the arctic and sub-arctic regions in the Nordic countries [21]. In northern Scandinavia and Finland, cloudberries have been significant both for Sámi nomads and settled peoples [22].

This article contains ethnobiological and ethnogastronomical perspectives [23, 24] and aims at highlighting the importance of cloudberry in Sweden as food in a diachronic and present perspective. Harvesting activities of wild berries for food in complex societies deal not only with micro-perspectives, with shared culture, linguistic and local knowledge perspectives, but must to a large extent also take into account macro-factors such as economic, legal, political and social structures and aspects, and society and its contexts in general [4, 25]. The results can contribute to expand our understanding of how local folk knowledge of wild plants is transmitted and transformed from traditional food systems into modern harvesting and cuisines. This study contributes also more globally to the discussion about the future of wild food products, especially for those who are promoting local small businesses, tourism and sustainable quality food [26], and as an extension, about the impact of commercialisation and climate change on wild foods and their roles in society and culture.

## Methods and sources

In order to understand the contemporary significance of foraging and utilisation of cloudberry, ethnobiologists must study the importance of it from both the historical and present perspectives [5, 10]. This study falls within the scope of historical ethnobiology, which is the study of the relationships between human cultures and the surrounding biota over time, focusing on how shared knowledge and practices regarding other organisms and ecosystems have developed and influenced cultural identity, resource management, and biodiversity conservation [27]. The approach is diachronic, meaning that it follows the development and growth of importance of cloudberries over time in a specific society and culture. In contrast to present-day ethnobiological studies, where interviews and data validation is possible, historical analysis must rely mainly on written materials and for the second half of the twentieth century possibly also on recordings with informants. Therefore, a full validation of data cannot be carried out and several of the methods used in modern ethnobiology are not applicable. We have employed a qualitative research design. The methods used in historical ethnobiology involve collecting and comparing data from different time periods to identify changes, patterns and causes. The choices of methods are primarily qualitative: we employ a combination of botanical, ethnobiological, ethnological, historical, cultural, social and linguistic methods to follow cloudberry over time [4, 10].

Using multiple sources is an essential research method among ethnobiologists [4, 28] and this is especially true for historical ethnobiologists: this study is based on ethnographic archive materials, local historical works, dialect plant name studies and studies of contemporary berry harvesting, preparation and consumption. For the historical background, we use records from the dialect and folklore archives, as well as botanical, ethnographical and sociological studies. For contemporary data, we analyse answers from 81 respondents to a questionnaire on “the use of wild edible plants” in Sweden in 2019, as well as participatory observation, discussions with other gatherers and personal experiences of gathering and preparation of cloudberry products. In the questionnaire we asked if the respondent gathered wild berries, and then: *Which berries? What do you make of them? Do you have a favourite berry?* The respondents consisted of 70 women, 7 men and 4 informants who did not specify their gender. A majority (*n* = 60) of the respondents lived in large cities in the southern part of Sweden. Only 10 respondents mentioned cloudberries. Most respondents lived in areas where cloudberries do not grow and data was therefore scarce. Cookbooks, newspaper articles and recipes and other printed works have also been utilised.

## Biology and ecology

**Fig. 2:**
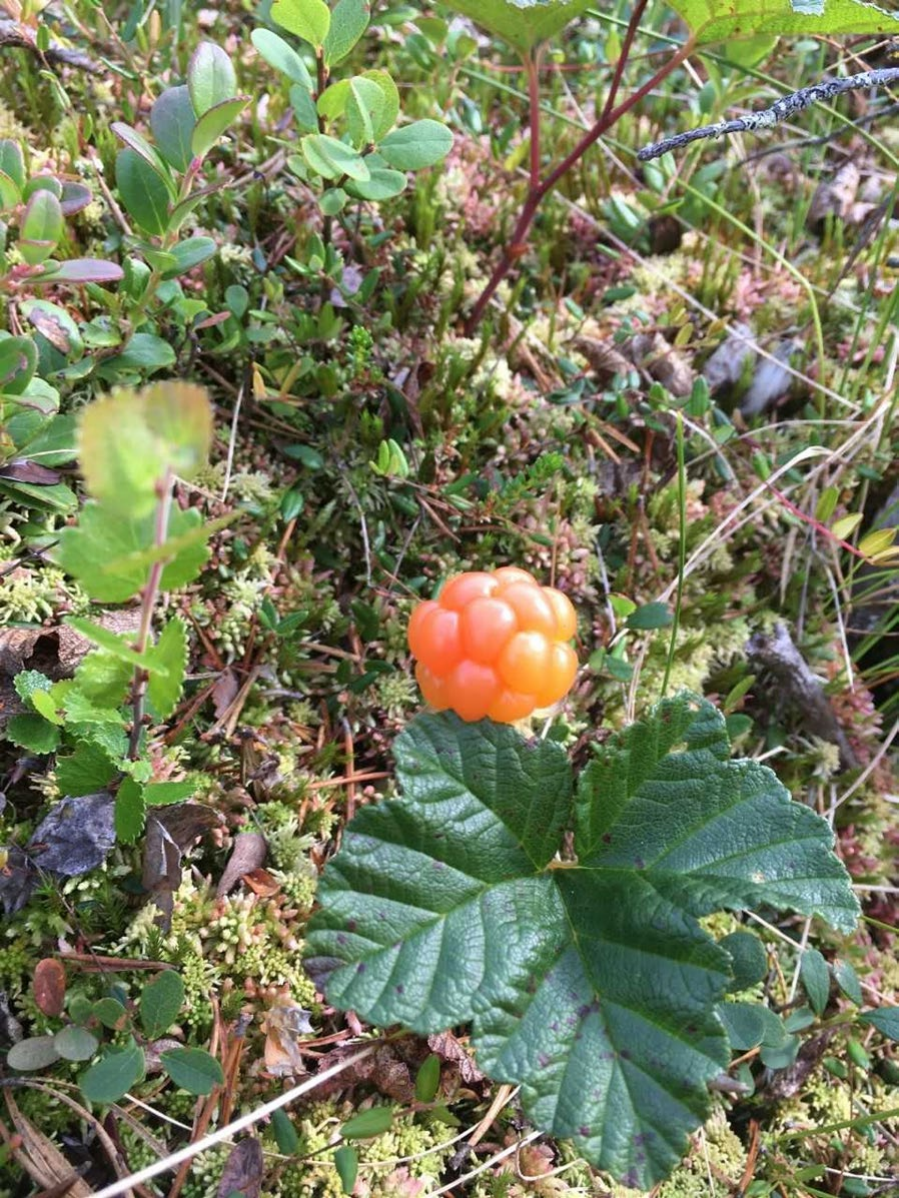
The amber-orange colour of the cloudberry fruit has given it epithets such as “gold of Lapland” or the “gold of the forest” (Photo Sonja Ek, Dala-Järna, Dalarna 2025)

Cloudberry, *Rubus chamaemorus* L., is a perennial, low-growing herbaceous shrub belonging to the Rosaceae family. It grows between 15 and 30 centimetres tall and has creeping rhizomes. The leaves are kidney-shaped and serrated with five to seven hand-like lobes. Flowers are large, solitary and white. Unlike other species of the plant genus *Rubus*, cloudberry lacks the ability to self-pollinate; it is dioecious, so male and female flowers are found on different individual plants [29]. The fruit contains vitamin A, C and E, phenolic compounds, quercetin and ellagitannins, which help combat oxidative stress and support immune functions in humans [30, 31] (Fig. 2).

**Fig. 3:**
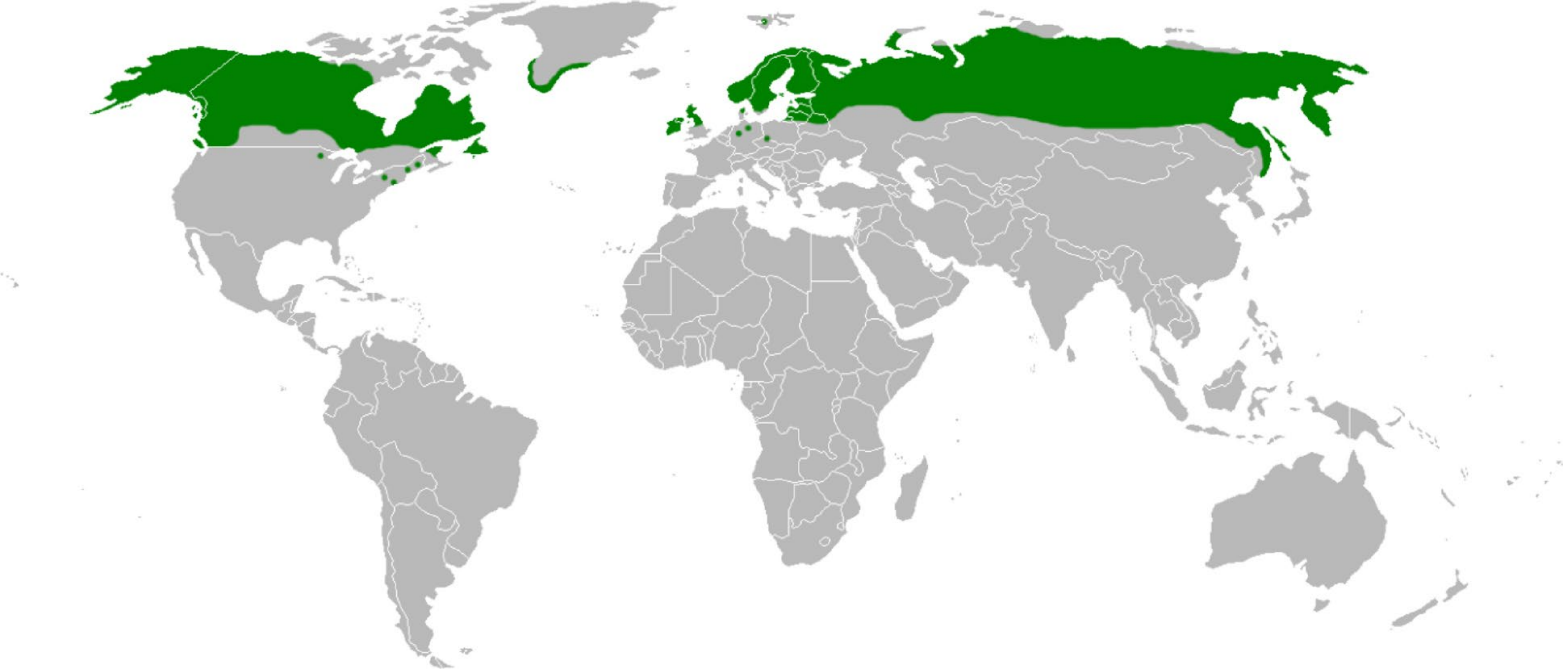
Circumpolar distribution of *Rubus chamaemorus* L. (Source Wikimedia Common CC BY-SA 3.0)

The cloudberry plant is a circumpolar and boreal plant, occurring naturally throughout the northern hemisphere in Eurasia from 78°N southwards to about 54°N and scattered to 44°N, mainly in mountainous areas, marshes and moors, and more frequently in the Baltic states as glacial relicts [32] (Fig. 3).

In North America, the berry grows from Canada to north USA, but it is now threatened in many areas [33]. The plant is cold-tolerant and can withstand low temperatures even to – 40 °C. The flowers are sensitive to frost, however: a frosty night during the flowering period in spring could damage flowers and prevent fruition. This makes harvests extremely difficult to predict. In some years, bogs are aglow with berries, while in other years they are almost empty. Cloudberries usually ripen in July-August in southern Sweden and later further north [18, 34]. Over most of the growth range, humans eat the berries traditionally. Many animals, such as bear, fox, capercaillie and black grouse also eat the berries, while the leaves are food for caterpillars of several Lepidoptera species [35].

## Study area: Sweden

Contemporary Sweden covers an area of 450,295 square kilometres, forming the eastern part of the Scandinavian Peninsula; Norway forms the western part. Nearly 69 per cent of the country is forest and woodland, and approximately 15 per cent of the total land area is located north of the Arctic Circle. Natural vegetation varies considerably due to various climate conditions and ecological settings, with mountains, forest regions, and coastal areas including archipelagos and agricultural landscapes. Eight vegetation zones can be distinguished: the boreal zone and its subzones cover most of the country [36].

Cloudberries grow in almost the entire country, from the mountain areas of Lapland in the north to the southernmost province of Skåne; only on the island of Öland it is absent. The Stockholm archipelago, for example, has plenty of small, rich cloudberry bogs. On the IUCN Red List, the plant is classified as Least Concern in Sweden [34]. Its importance as an economic food plant for the inhabitants is reflected in many toponyms with the prefix ‘cloudberry-’ (*Hjortonberget*,* Hjortronkärret*,* Hjortronmossen*, *Hjortronbärsmyren*, etc.) for bogs, mires and hills all over Sweden [37].

Minorities in Sweden connected with cloudberry gathering include several groups of indigenous Sámi in the far north. The Sámi (population 20,000–40,000) have a background in nomadic lifestyles and as fisher-gatherers [38]. Also groups of Finnish-speakers, including the linguistically assimilated Forest Finns in central Sweden and the Meänkiele-speaking Tornedalians with 70,000 native speakers, a border minority in the northernmost part of the country, traditionally use cloudberries [39]. Nowadays in Sweden there is an estimated 20.3 per cent of the population with a foreign background, who have arrived in the country since the end of World War II: some of them are also foraging berries, wild plants and mushrooms [9]. The majority of the Swedish inhabitants are urban: about 89 per cent of the population lives in towns and cities [36].

## Results and discussion

### Local names

Commonly known as *hjortron* or *multer* in contemporary Swedish, the cloudberry and plant have been known by a number of local names in various parts of Sweden. The oldest names are recorded by Johannes Franckenius in 1638 and 1659 and are still in use: *hiortron*, *molter* or *multerbär* and *myrbär* [40, 41]. The folk names have been discussed by several well-known dialect researchers and plant name experts such as Karl-Hampus Dahlstedt [42] and Kjell Furuset [43].

Cloudberry was called *myrbär* ‘bogberry’ in central Norrland. Further west it was called *multa* or *molta*, and in the coastal areas of this northern province, *snottra* or *snotterbär*. Overripe berries have in some provinces been called *blötbär* ‘wet berry’ (Härjedalen. Jämtland, Ångermanland, Lapland), *blötgubbe* (Uppland, Gästrikland, Västmanland, Dalarna), *blötlaska* (Gästrikland), *blötmulta* (Värmland, Jämtland), *blötsnottra* (Medelpad, Ångermanland, Västerbotten, Norrbotten), *laska* (Gästrikland) or *soppa* (Sörmland, Närke, Östergötland). Some authors state that overripe berries were juicy and particularly tasty, and in folk taxonomy, a distinction between ripe and unripe berries was made. The unripe ones were referred to as male or ‘old men’, *hjortagubbe* (Östergötland) or *hjortongubbe* (Uppland), while ripe berries were female or ‘old woman’, *blötgumma* (Uppland), *gumma* (Småland, Sörmland), *hjortakäring* (Småland, Östergötland), *hjortrakäring* (Östergötland), *hjortronkäring* (Sörmland), *käring* (Skåne, Småland, Halland, Västergötland, Östergötland, Sörmland), *käringemulta* (Dalsland), *smörkäring* (Östergötland) [44, 45]. In the Övdalian language in northern Dalarna the berry is known as *moirbär* ‘bogberry’ [46].

In Meänkieli, the Finnic language spoken in the northernmost part of Sweden, particularly in the Torne River Valley, the berry and plant are known as *hilla*, *lyömänä* and *lakka* [47]. Similar phytonyms are also known in Finnish: *hilla*,* lakka*,* muurain* and *suomuurain* [48]. In North Sámi, it is known as *luomi*, in Lule Sámi as *làttak*, in Pite Sámi as *láddak*, Ume Sámi *láddage* and South Sámi as *laadtege* [49, 50].

### Historical uses

In the circumpolar area, cloudberry was probably the most important berry for humans in pre-industrial societies. For the Inuit in Canada and Alaska, as well as among Chukchee and Koryaks in the Siberian Far East, it was an important source of vitamin C [22, 51]. Considerable quantities of cloudberries are still harvested for human consumption in the Nordic countries, Russia and the northern areas of North America [52, 53]. Cloudberry plants probably spread north after the last large-scale Ice Age, and has certainly been gathered and consumed on the Scandinavian Peninsula ever since hunter-gatherers moved northwards from continental Europe. There are so far, however, no archaeobotanical finds or written data available from pre-historic or even up to medieval times [54].

**Early modern period (sixteenth to eighteenth centuries)**: The oldest known written evidence from Sweden for preserved cloudberries is found in accounting documents under the name *syltade hiortron* ‘preserved cloudberries’ from Gripsholm Castle in 1549 [54, 55]. By the seventeenth century, physicians knew the importance of cloudberries for preventing scurvy [56]. The berries have also been attributed with medicinal properties: Carl Linnaeus recommended them as cures for dysentery, haemoptysis, pneumonia and especially scurvy [57, 58].

Cloudberry is one of very few berries traditionally used as food before cheap sugar became widely available in the Nordic countries; most other berries were only a sour and on-the-spot snack for children [9]. Reasons why berries were not utilised as food in pre-industrial and pre-sugar Swedish society is that it usually required more energy and time to collect berries than the nutrition the berries could provide, and they could not be easily preserved for the winter except in the far north where summers are very short [9]. Therefore the use of cloudberries in households remained scarce before the nineteenth century [54].

Only in northernmost Sweden, cloudberries were an important dietary element for the Sámi. In the 1670 s, the priest Nicolai Lundius noted that Sámi women are very skilled in gathering berries in the summer, especially cloudberries [59]. His contemporary, Samuel Rheen, reported that cloudberries were simmered slowly in a cauldron in their juice, then a pinch of salt was sprinkled on them and the berries placed in small boxes made of birch bark. These were buried in the ground and covered. In winter, the boxes were dug up and the contents eaten. The berries preserved in this way had a fresh and good taste. Barrels with berries could also be lowered into cold water springs [60]. Sixty years later, Linnaeus noted that the Sámi “eat with delight the berries mixed with reindeer milk, and I too am not averse to this delicacy of theirs, which is more harmless than confectionery and other sweets [61]. They store whole berries, buried in snow, throughout the winter and eat them in the spring, [and the berries are] just as tasty as when they were buried.” Cloudberries are still an importand food item among the Sámi in the north as well as among Tornedalians [23, 62].

#### Modern period (end of eighteenth to beginning of the twentieth century)

In his survey of the Swedish peasant diet in the nineteenth century to early twentieth century, ethnologists Nils Keyland’s testify to the importance of the berries at this time in rural households. Cloudberries were highly valued among the natural resources consumed by the peasantry of northern Sweden; from Hälsingland, Keyland described *myrbärsgröt*, a raw dish made from cloudberries and flour. Cloudberries, like other berries, could also be used as an ingredient in pancake dishes. The berries were considered festive food and could therefore be served with cream. Little variation in how the cloudberries were used is mentioned [9, 15]. Forest ecologist Lars Kardell concludes, after reviewing historical data, that cloudberries had no energy or economic importance for the peasants, but the berries were valued as a remedy for deficiency diseases [63].

The oldest published recipe in Sweden for pickled cloudberry dates to 1743. Around this time the oldest cookbooks refer to eating the fruits in a preserved state. When sugar became more common in the nineteenth century, cloudberry jam recipes appear more often [64]. In 1896, the famous cookbook author Charles Emil Hagdahl published a detailed jam recipe for boiling and then pouring the berries into jars “which are left to stand overnight, then covered with waxed paper and tied up” [65].

**Fig. 4:**
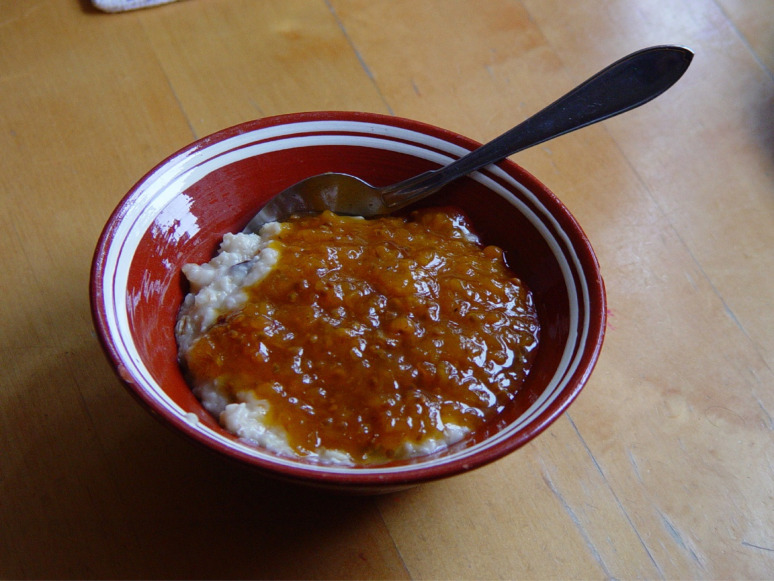
Oatmeal porridge with cloudberry jam (Photo Ingvar Svanberg 2008)

Peasants in many parts of Sweden did not harvest berries in pre-industrial times, partly because they lacked preservation methods and more importantly, the berries ripened at the same time as grain. The farmers were busy with other economic activities including hay-making [54, 66]. The situation in the cold north was different: one author from the northern province of Jämtland explained: “Only cloudberries were valued in the old days [among all berries], but they were collected much more in the years when they were in abundance.” The berries were boiled without sugar into a porridge, which was considered more as a substitute for grain-based porridge than a “real” porridge Fig. 4 [67, 68]. It was not unusual for rural inhabitants to stock up on a whole barrel of berries for winter food [69]. Pounded or mashed cloudberries were in some regions stored in wooden barrels. Jokkmokk settlers consumed cloudberries fresh as long as the berries were available, and a few kilos would be put into a well or cold spring for some time [22, 70].

#### Industrialisation (twentieth century)

**Fig. 5:**
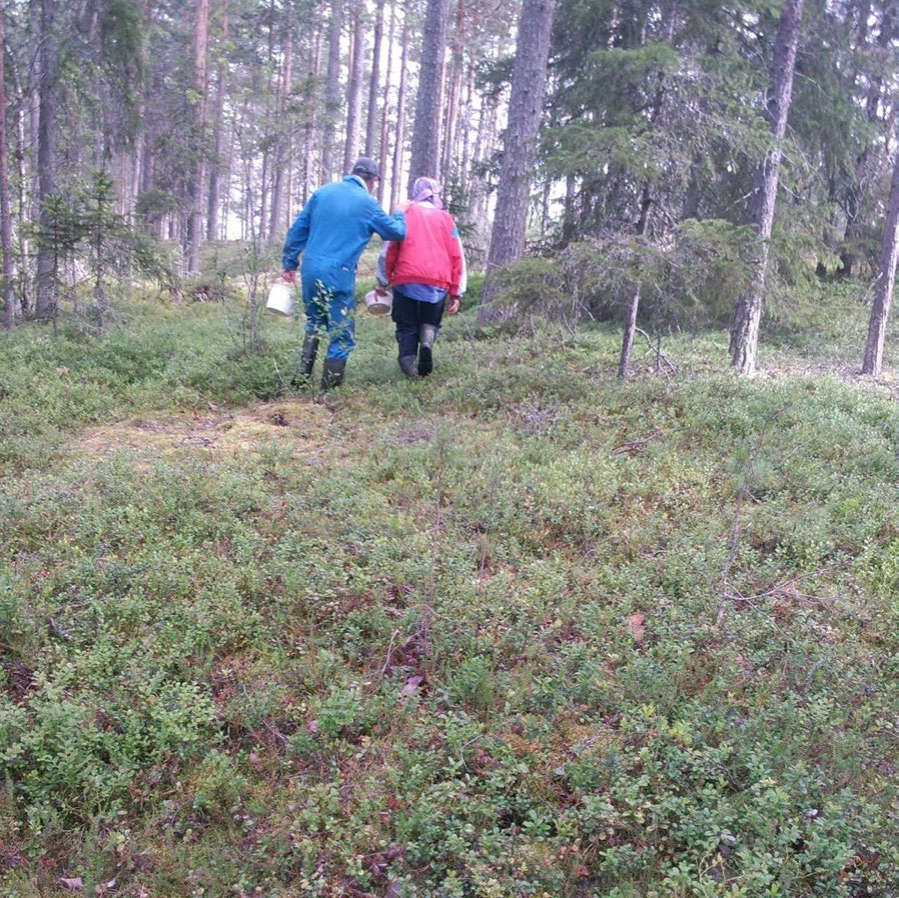
Picking cloudberries is still an important leisure activity for many people in northern Sweden. Sangis, Norrbotten (Photo Anna-Lena Forsberg)

Wild berries in general, including cloudberries, became more popular as sugar became cheaper. Propaganda in favour of consuming the vitamin-rich berries for public health purposes also played a role [9]. During World War II, there was a shortage in Sweden of various foodstuffs due to import difficulties. Several initiatives to increase food supplies focused on wild food. Local tourist associations, among others, encouraged gathering and consumption of cloudberries. This engaged especially urban dwellers. Until the 1960 s, selling self-gathered cloudberries was an important source of income for many people living in rural areas in northern Sweden. This changed over the following decades with TV cooking shows, magazine food articles and cookbooks increasingly exploring local foods. In 1995, some 3.8 million of litres were estimated to be gathered for home use by household gatherers [52, 71]. Although harvesting cloudberries can be hard work, spending time in the forest, hills and bogs collecting berries also has a strong recreational value, which is still valued highly by hobby gatherers. Figure 5 Of our 81 survey responses in 2019, ten stated that they ate cloudberries; all were from northern Sweden. The majority (*n* = 9) gathered personally and prepared sweetened jam. One person stated that they froze the berries.

## Local folk knowledge, norms and practices

There are many beliefs and traditions associated with the flowering of the plant, most of which have now been forgotten. The flowering signalled spring and was perceived as a forecast for the upcoming harvest in many provinces. “When the cloudberry blooms, the roaches spawn” was a saying in Lycksele, Lapland. If it was starry on New Year’s night, there would be plenty of cloudberries in the summer, it was told in Härjedalen; the same was said about the Christmas night in the province of Värmland. In Arjeplog, Lapland, it was believed that if it snows a lot on Good Friday, there will be plenty of cloudberries. When hunting, gathering berries, etc., one was not permitted to say “good luck” as this brought bad luck; this is still a general superstition throughout Sweden [72].

Common knowledge, shared by local people, about where, when and how to collect cloudberries are still important today. This also involves observations, practices and norms that foragers must consider and carry out. The berries are sensitive to frost, driving rain and drought, and the harvest season is quite short. Gatherers must observe the environment and the weather over a longer period in order to know when the time and berries are ripe; it takes only about two weeks until the berries become overripe and unfit for gathering. According to interviewees, local foragers often keep the best bogs with large numbers of ripe cloudberries a private secret; only parked cars along the roadsides reveal where gatherers have entered the forest. Foragers also respect other people’s cloudberry patches and do not go there when someone else is gathering; those places are usually known only to the locals. It is often necessary to walk long distances to reach a good spot [73]. There are records of families having special nicknames for their favourite places. ‘God’s cabbage patch’ (*Guds kålgård*) is recorded from Dalarna in the late 1930 s [74].

**Fig. 6:**
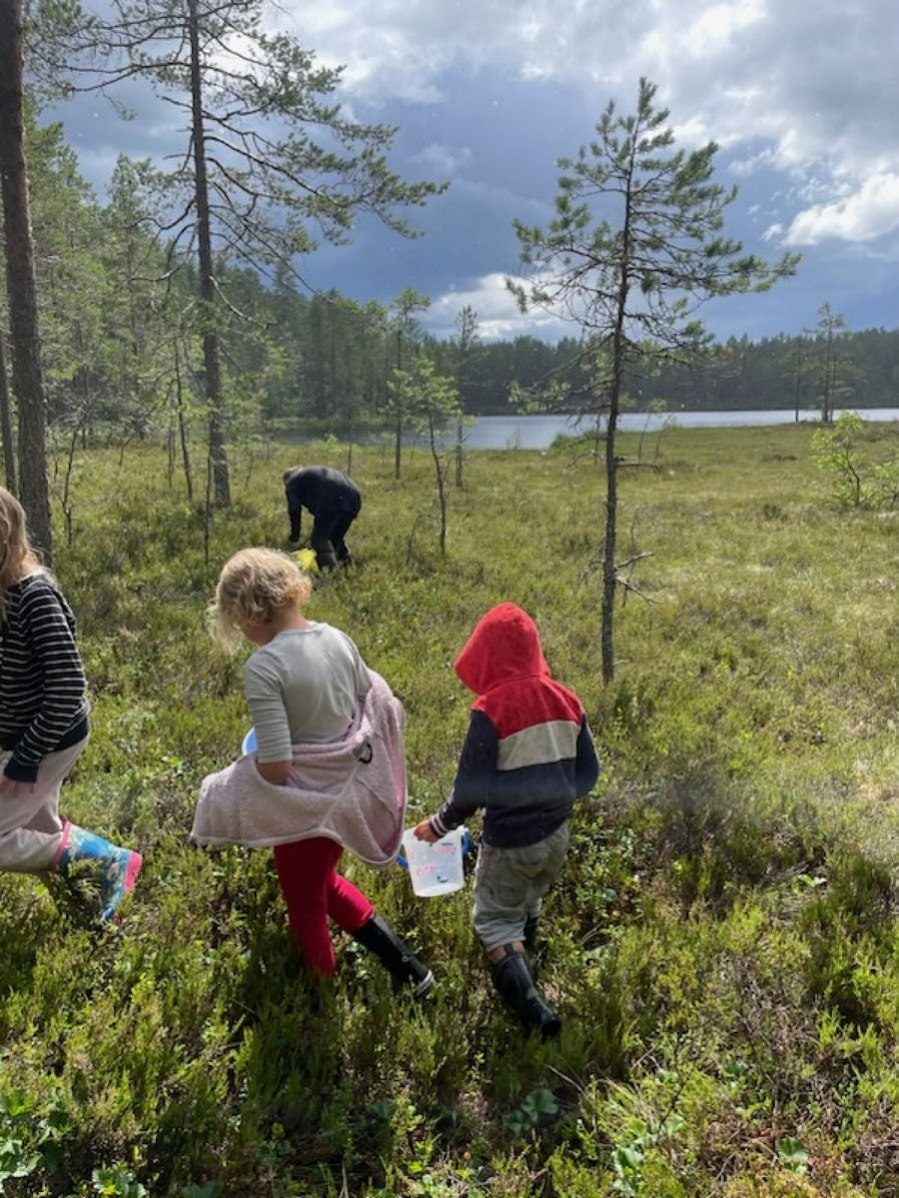
Children also participate in picking berries from an early age. A bog in Dalarna (Photo Isak Lidström, 2024)

**Fig. 7:**
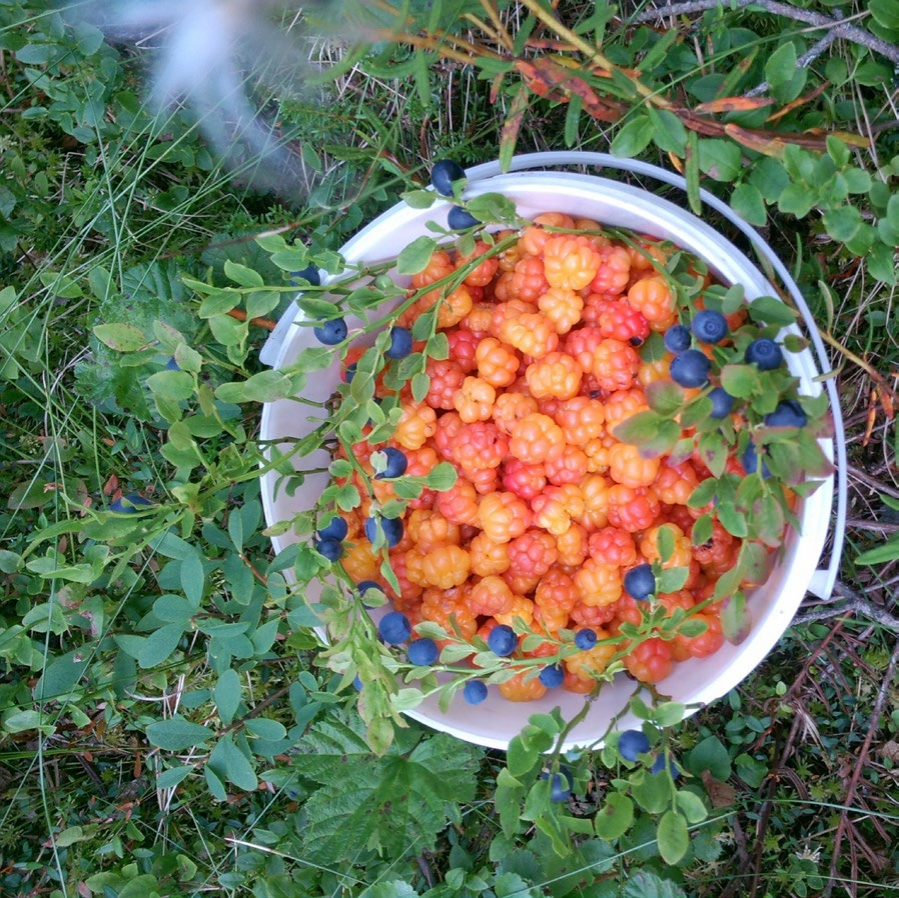
A bucket with harvested cloudberries and branches of bilberries. From Sangis, Norrbotten (Photo Anna-Lena Forsberg)

**Fig. 8:**
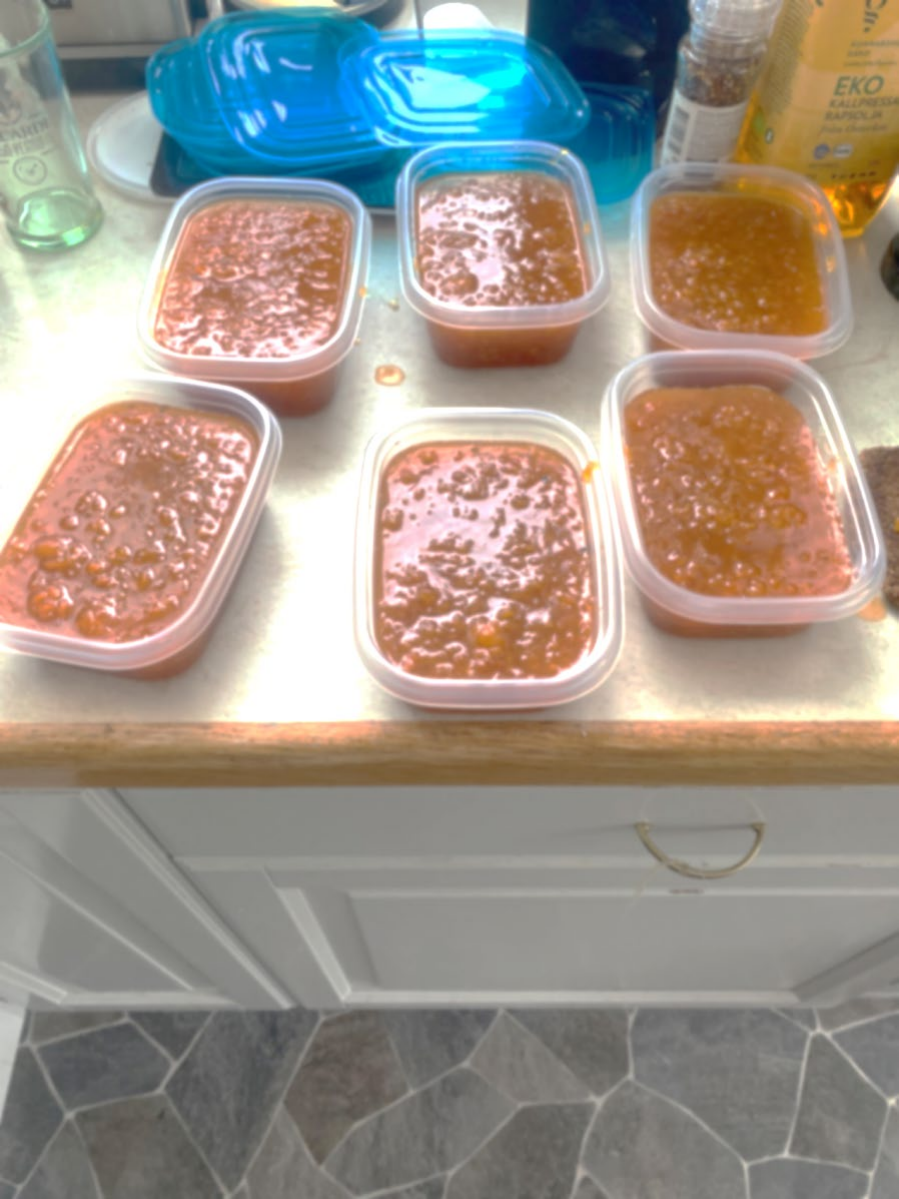
Freshly made cloudberry jam, packed and ready for the freezer (Photo Isak Lidström, 2024)

The soft and wet terrain may be difficult to manage and it is potentially dangerous for people who are unused to it. Figure 6 Every year, cloudberry foragers get lost in the forests and mountains and are only found after intense searches. There have even been cases of cloudberry foragers being attacked by bears or walking into a water-filled bog and having to be rescued. Rubber boots, a backpack with food and drink, and effective mosquito repellents are also necessary attributes for foragers. The special ecological conditions that prevail in the northern marshes mean that collecting cloudberries can be a demanding task which requires skills and knowledge. Berry enthusiast Örjan Armfelt Hansell offers a dramatic description: firstly, many hardships and trials await cloudberry foragers. Gathering cloudberries in the heat (25 °C is considered hot weather in the north), with no wind but millions of horseflies and mosquitoes, and billions of midges trying to distract the collector is an experience “like nothing else”. An early frost can also ruin the entire harvest; thus, there are no guarantees at all for success [18] ( Figs. 7 and 8).

### Commercial harvest

There is no ban on commercial large-scale berry harvesting in Sweden, as long as it does not cause any loss or inconvenience to landowners, other than the reduction of berries in their forests [75]. The most common and commercially significant species are bilberry and cowberry, whose bushes cover nearly a fifth of Swedish forests and in some years produce over a million tonnes of berries [76]. Other wild berry species are harvested in smaller quantities; cloudberry is at present the commercially third most significant berry in Sweden [9].

In Norway, cloudberries are mentioned as an export product as early as the sixteenth and seventeenth centuries [77]. The berry trade from northern Sweden to Stockholm began in the seventeenth century [78]. In 1737, Carl Linnaeus noted that a large quantity of pickled cloudberries was sent annually from the northern province of Västerbotten to Stockholm [61]. From the 1770 s onwards, newspaper advertisements appear for pickled cloudberries sold in barrels in the Swedish capital. Cloudberries continue to be an important element in the economy of the Sámi, settlers and mountain peasants in the far north of Sweden [79].

**Fig. 9:**
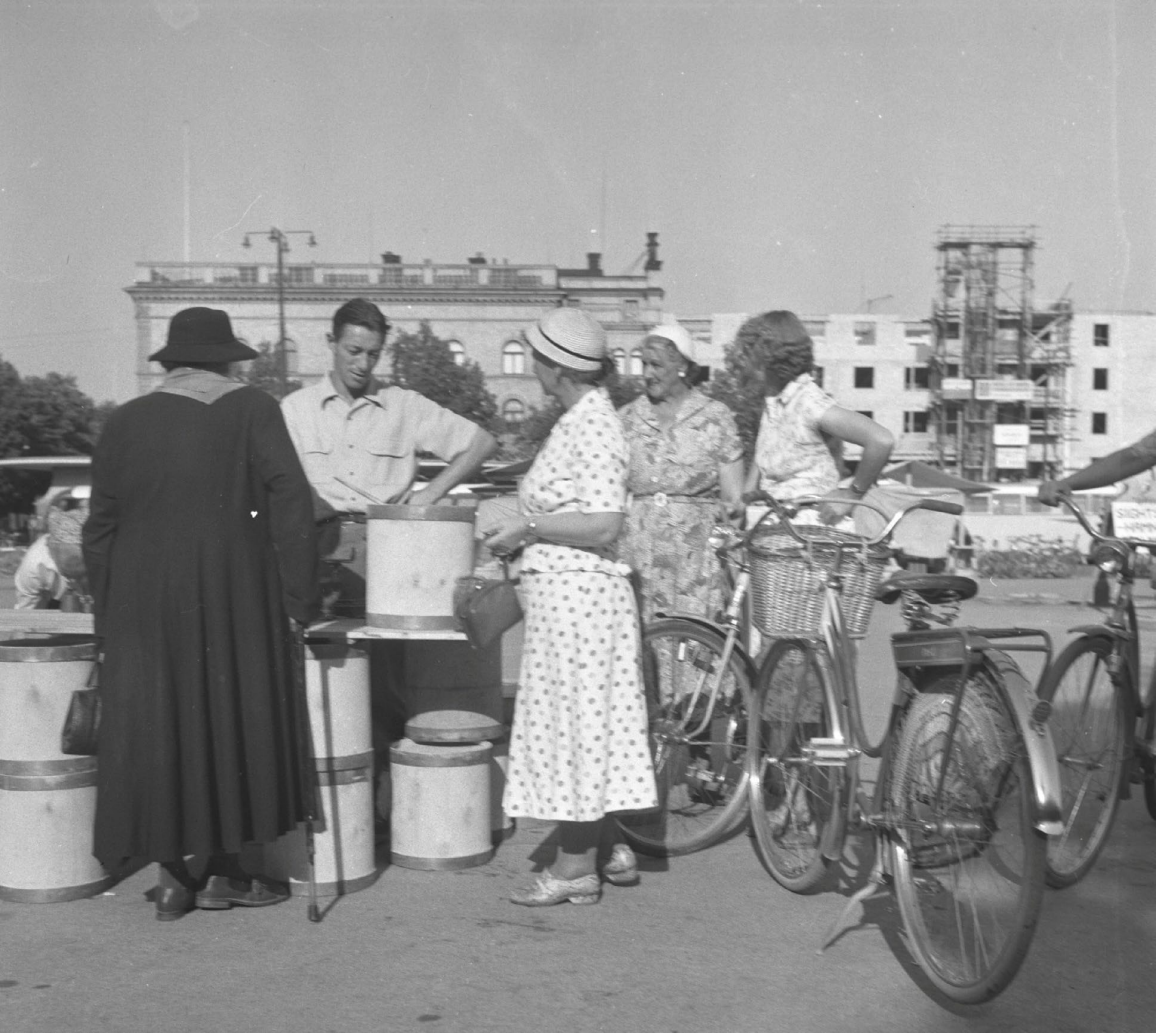
Sale of cloudberries stored in wooden barrels in Gävle square in August 1953 (Photo Carl Larssons Fotografiska Ateljé AB, Gävleborg Museum, PDM)

**Fig. 10:**
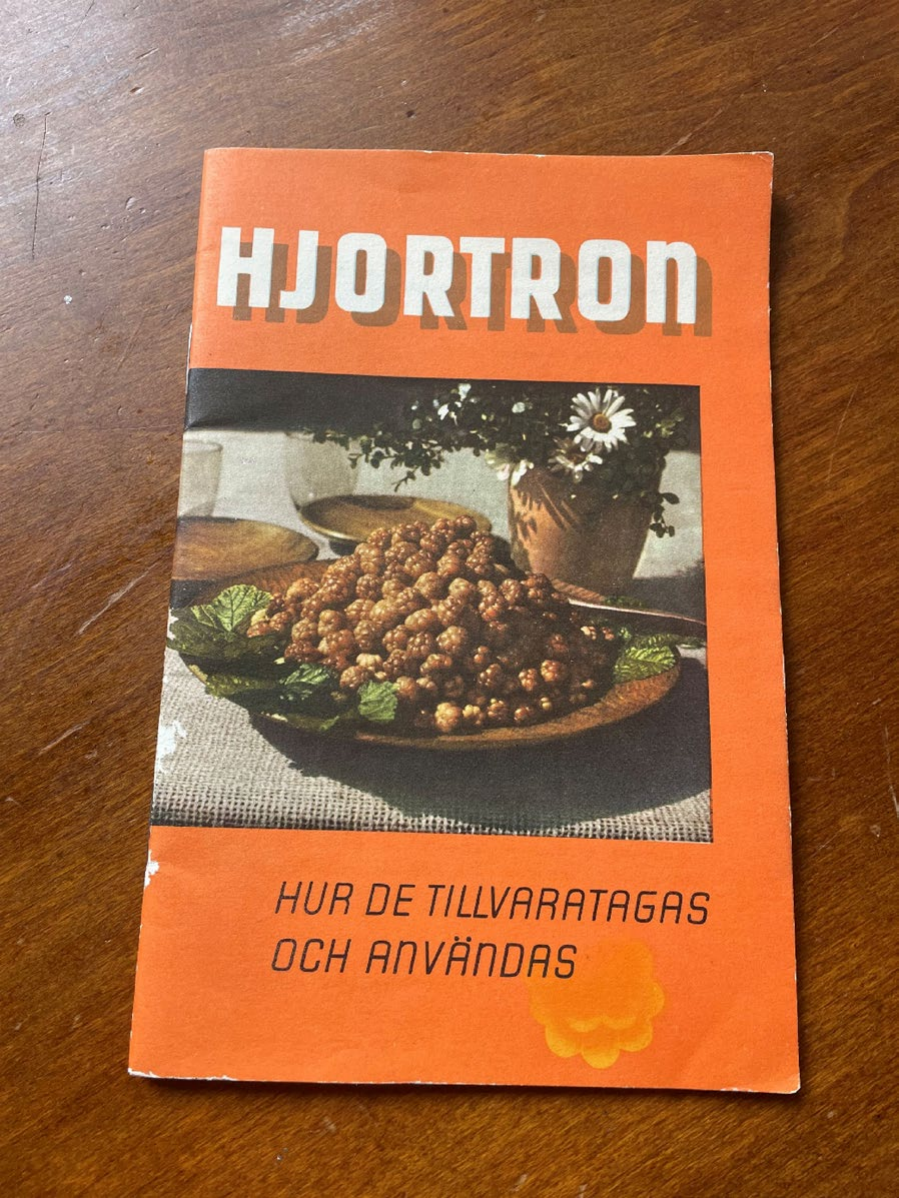
Booklet published in 1939 by *Svenska Sockerfabriks Aktiebolaget* (Swedish Sugar Company), with recipes, to promote use of cloudberries as food (Photo Navarana Ingvarsdóttir Olsen)

When imports of citrus fruits was reduced due to World War II, the Swedish authorities encouraged increased consumption of local vitamin C-rich fruits such as cloudberries, rose hips and also less used berries such as rowanberries (*Sorbus aucuparia* L.), which consequently stimulated both harvest and sales in urban areas. Figure 9 Special crisis committees were established locally: their main purpose was to hand out rationing cards and distribute fuel, food, animal feed, etc., but they also promoted berry gathering. In 1940, lectures were offered about how households could make use of cloudberries, for example. In Vilhelmina, Lapland, a cloudberry winery (*musteri*) was established to make more efficient use of cloudberries [80]. In 1939, before the outbreak of World War II, the Swedish Sugar Factory Ltd (*Svenska Sockerfabriks Aktiebolaget*) published a small booklet promoting increased utilisation of cloudberries and providing recipes for their use Fig. 10 [81]. This continued the tradition of an earlier cookbook published during World War I, encouraging households to make greater use of cloudberries in difficult times. The berries could be used to make jam, juice and compote [82]. During the war, also tea substitutes made from local plants were sold, including leaves from cloudberry, wild strawberries, rosebay willowherb and other plants [83] (Fig. 10).

Due to improved economic conditions and changes in culinary practices and tastes after World War II, and increased imports of fresh vegetables and fruits and the wider distribution of supermarkets, interest in harvesting berries declined during the 1970 s and 1980s. This occurred despite a growing interest in local wild foods and the introduction of berries into Nordic fine dining. At the end of the 1970 s, only five to seven% of all berries were harvested in Sweden. In a 1977 survey, people were asked how much they had gathered for private use during the year. Households collected a total of approximately 40,000 tonnes of berries at this point in time. The same question was asked in a survey twenty years later: the amount had by then decreased to around one-third of the previous [67].

The harvests of Swedish cloudberries were often too small to satisfy the needs of the jam industry, which is why berries were imported from Finland and the Soviet Union. The berry industry also became increasingly dependent on seasonal workers from abroad: workers from Eastern Europe, mainly from Poland, began to be recruited in the 1980 s, and they arrived in Sweden on tourist visas. After the fall of the Soviet Union in 1991, the numbers of gatherers from the Baltic states Estonia, Latvia and Lithuania increased. The trend remained unchanged for several years but declined as workers from Eastern Europe found other, more lucrative and less challenging jobs in other European countries or at home. Since the 2000 s, most seasonal workers are from Southeast Asia, mainly Thailand, Vietnam and Cambodia. The behaviour of the Thai pickers are different from the rural Swedish foragers: they have often been rice farmers and are used to hard work, starting to gather at four in the morning (the sky is already light in the north at this time of day). and after an intense twelve-hour workday bring back some 40 L. They are persistent pickers in the berry forest, but the local knowledge of the cloudberries must be provided by their supervisors. Thai women with permanent residence in Sweden also gather and sell cloudberries [9, 84]. Probably they have learnt gathering from their Swedish spouses.

After several high-profile cases of workers being exploited in recent years, stricter requirements and control on recruitment agencies have led to a drastic reduction in the number of seasonal workers; the numbers are down from 5,000 before the scandals to 1,200 in 2024, and only around 90 Thai workers were expected in 2025 (Table 1).

**Table 1:** Seasonal berry-pickers workers in Sweden

Year	Granted work permits for berry-picking
2015	3 990
2016	3 326
2017	3 070
2018	3 987
2019	6 199
2020	3 092
2021	5 111
2022	6 594
2023	5 372
2024	1 272
2025	89

https://www.migrationsverket.se/

Other workers have not been found to replace the efficient Thai gatherers, and therefore the total volume of commercial collection will inevitably decrease, not only temporarily but most probably also long-term. The berry industry will be provided with smaller quantities of cloudberries, leading to shortages in the production of jams and other products, and increased imports and prices [85]. Efforts to import berries from neighbouring countries are being carried out, but for instance Finland also has a cloudberry industry and large consumption of the berry. Since the 1990 s, there are attempts to establish cloudberry as a cultivated plant, and in the 2000 s some cultivars have been published, but success is still expected [86].

### Contemporary cuisine

**Fig. 11:**
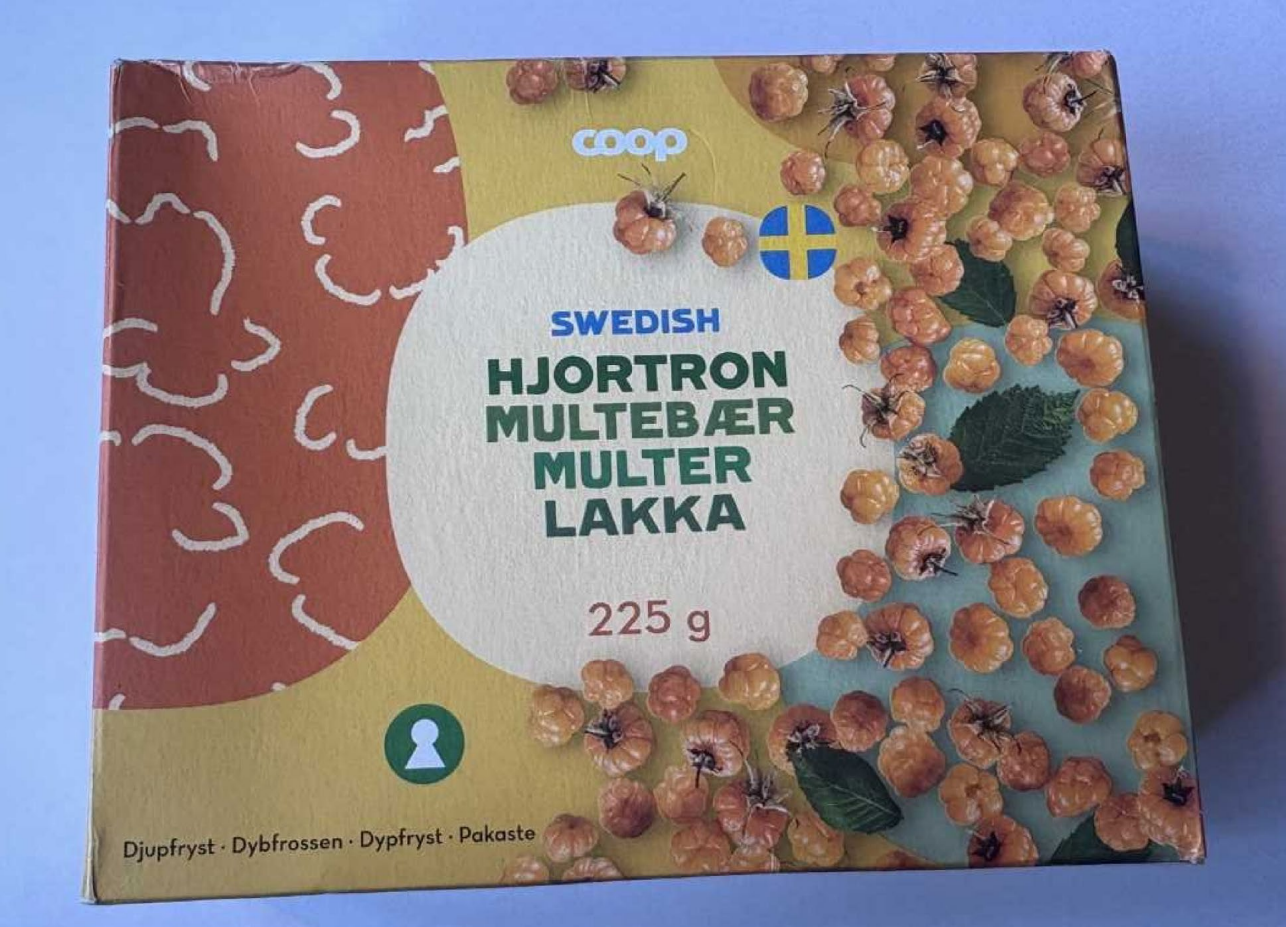
Frozen cloudberries are available in grocery stores and supermarkets all over Sweden (Photo Navarana Ingvarsdóttir Olsen, November 2025)

**Fig. 12:**
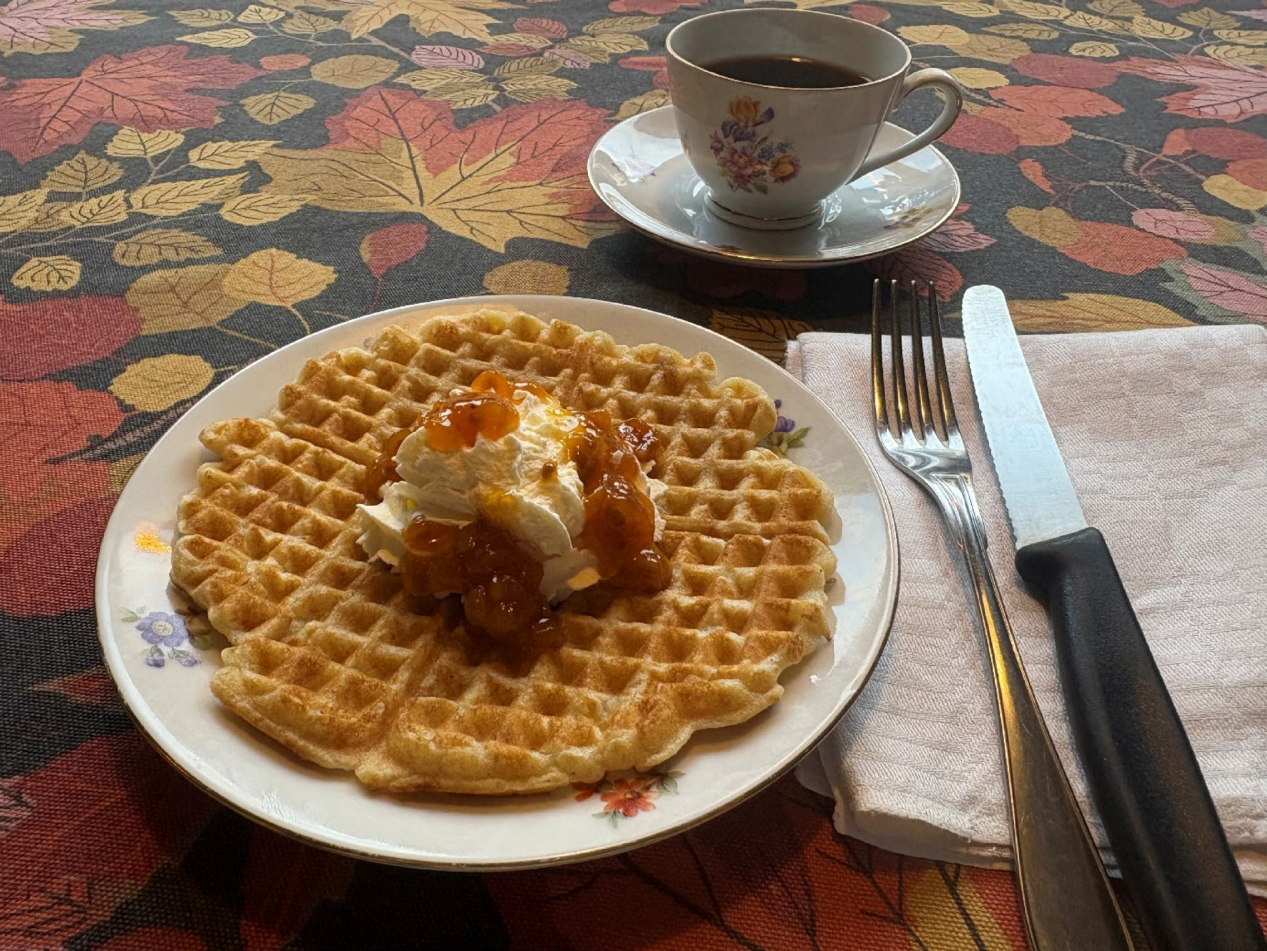
Waffle served with cloudberry jam is a common dessert in Sweden (Photo Navarana Ingvarsdóttir Olsen, November 2025)

Cloudberry became popular and has maintained an overall importance in contemporary Swedish cuisine for some seventy decades. Figure 11 It is mostly known as jam to a broader audience: the widely used cookbook *Bonniers Stora Kokbok* (1983), for instance, only mentions cloudberry sweetened jam [87]. Waffles with whipped cream and cloudberry jam – a festive dish served at parties – is an appreciated urban treat and often sold at festivals, fairs and events all over the country, as well as at the Skansen open air museum in Stockholm [9, 88] (Fig. 12).

Cloudberries are today considered an exclusive delicacy and connected to festive occasions or celebrations in Sweden. Bringing a jar of cloudberry jam or a bag of frozen berries as gifts to friends and relatives who lack access or opportunity to gather themselves is highly appreciated, as several of our informants have emphasised. Since the introduction of cloudberry into fine dining restaurants in the 1980 s and 1990 s, they can be found in a wide range of both sweet and savoury dishes and contexts. Creative food artisans, cookbook writers, chefs and TV food show hosts have developed new dishes and products based on cloudberry. The berries are mainly eaten fresh or frozen and are particularly popular in desserts such as sweetened cloudberry jam with vanilla ice cream, cloudberry parfait (mentioned for the first time in 1955), cloudberry marmalade (since 1951), cheesecake (since 1997) and spicy chutney. The berry is used to make jelly, liqueurs, sauces, various desserts (mousse, compote, etc.) and pastries, and has found many other innovative uses especially in luxury restaurants. In fine dining, cloudberry sauce adds flavour and luxurious taste to meat, game or fish, and a dessert of cloudberry mousse with crème caramel can be enjoyed in many restaurants. The leaves can be brewed into tea. A small recipe collection published by *Sockerbolaget* (The Sugar Company) in 1991 recommends making cloudberry parfait, liqueur and a special dessert made of cloudberries, almond paste and whipped cream [89].

Regional specialities, such as the traditional milk dish from Torne River Valley, *leipäjuusto* ‘bread cheese’, is eaten with cream and cloudberry jam; this dish is popular in Finland as well. A sweet-savoury dessert, popular since the 1970 s, is warm Camembert or Brie cheese with cloudberry preserve; highly popular is also warm cloudberry jam and ice cream, or cloudberry jam with Swedish ginger snaps (*pepparkakor*). The thin ginger snaps used to be eaten only for Christmas but are today available all year round; ginger snaps or their taste are now being combined with all kinds of foods and drinks including mineral waters. This developed use of cloudberries reflects changing dietary habits and willingness to include new flavours in the diet, which food researchers identify as a typical feature of the modern, internationalised and constantly changing Swedish cuisine [90].

**Fig. 13:**
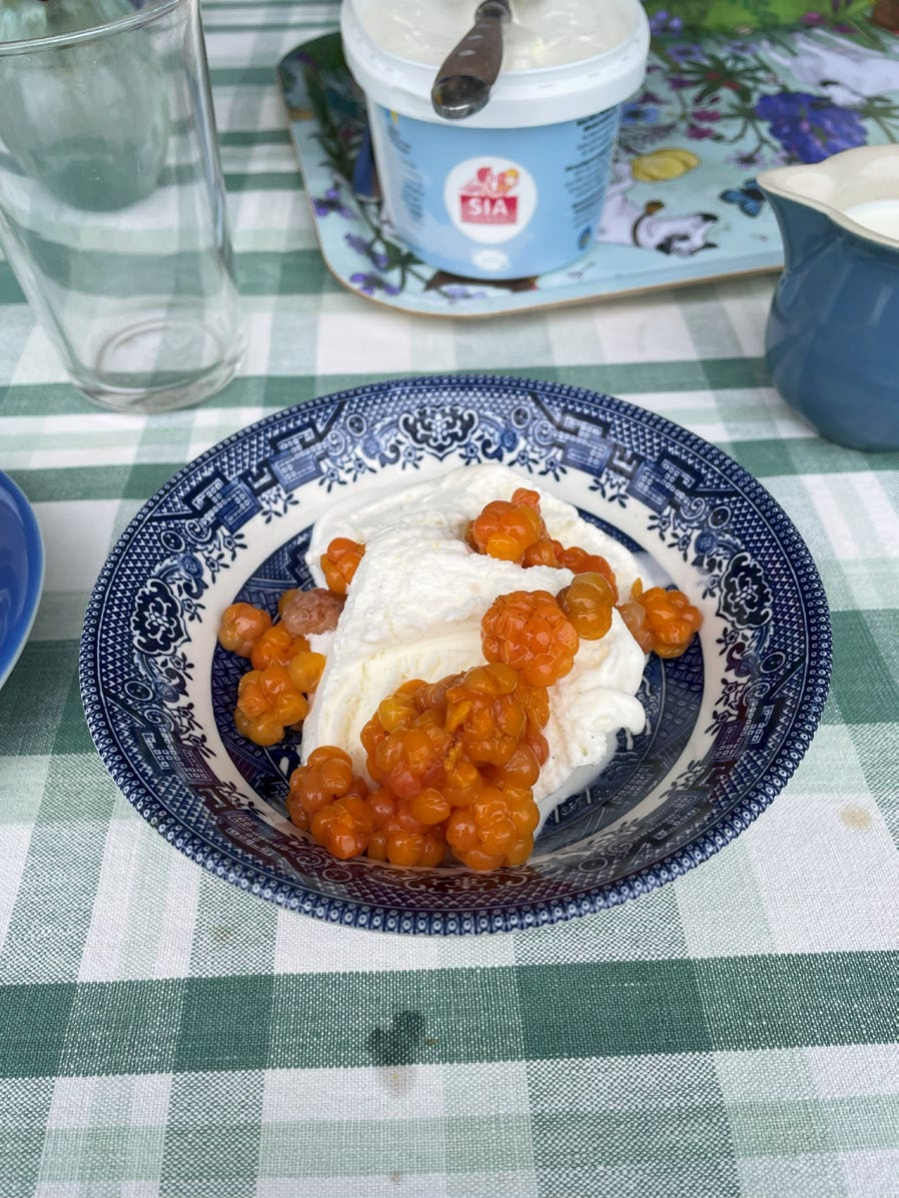
Vanilla ice-cream with macerated cloudberries (Photo Ingvar Svanberg, July 2025)

A survey among a small number of informants (*n* = 12) in the summer of 2025 yielded a great variety of answers to the question: *How are you consuming your cloudberries this year?* Several respondents explained that cloudberries were used as a luxurious jam on waffles, or served for dessert, hot or cold on ice cream or whipped cream. Figure 13 Others said they had cloudberries with yogurt or porridge for breakfast. A couple of respondents told that they mixed cloudberries with *crème fraîche* or sour cream and served the sauce with smoked salmon. It was called *myrens dröm* ‘the bog’s dream’ and probably originated from a magazine recipe, confirmed by the fact that it was found in several recipes on the internet. A pizza parlour in Vemdalen, Härjedalen, has come up with an original and innovative way to serve cloudberries: *Pizza Rudolf* is a pizza topped with reindeer meat, chanterelles and cloudberries.

In *haute cuisine*, wild berries including cloudberries keep to a special niche, especially in the northern part of Sweden, where cloudberries are marketed as a regional speciality [66]. Cloudberries are often used in dishes served at Swedish official occasions as well [91]. They are an obvious ingredient on tables when representing the country internationally: one typical example is the Nobel Prize dinners, where *Parfait aux mûres de Laponie*, Lapland cloudberry parfait with almond flakes (1956), *Savarin aux fausses-mûres avec crème moka*, cloudberry savarin with mocha cream (1970), *Mousse d’amandes aux mûres arctiques*,* sorbet au lait d’amandes*, almond mousse with Arctic cloudberries and almond milk sorbet (2004), *Nuage de sudachi*,* sorbet aux mûres polaires*,* miettes de miso et feuille de riz en friture*, cloud of sudachi fruit, polar cloudberry sorbet, miso crumbs and deep-fried rice paper (2016) have been served. Desserts and other dishes featuring cloudberries are also served at international trade fairs and at Swedish embassies worldwide [92]. Neighbouring Finland also includes cloudberries regularly when presenting or representing the country abroad.

**Fig. 14:**
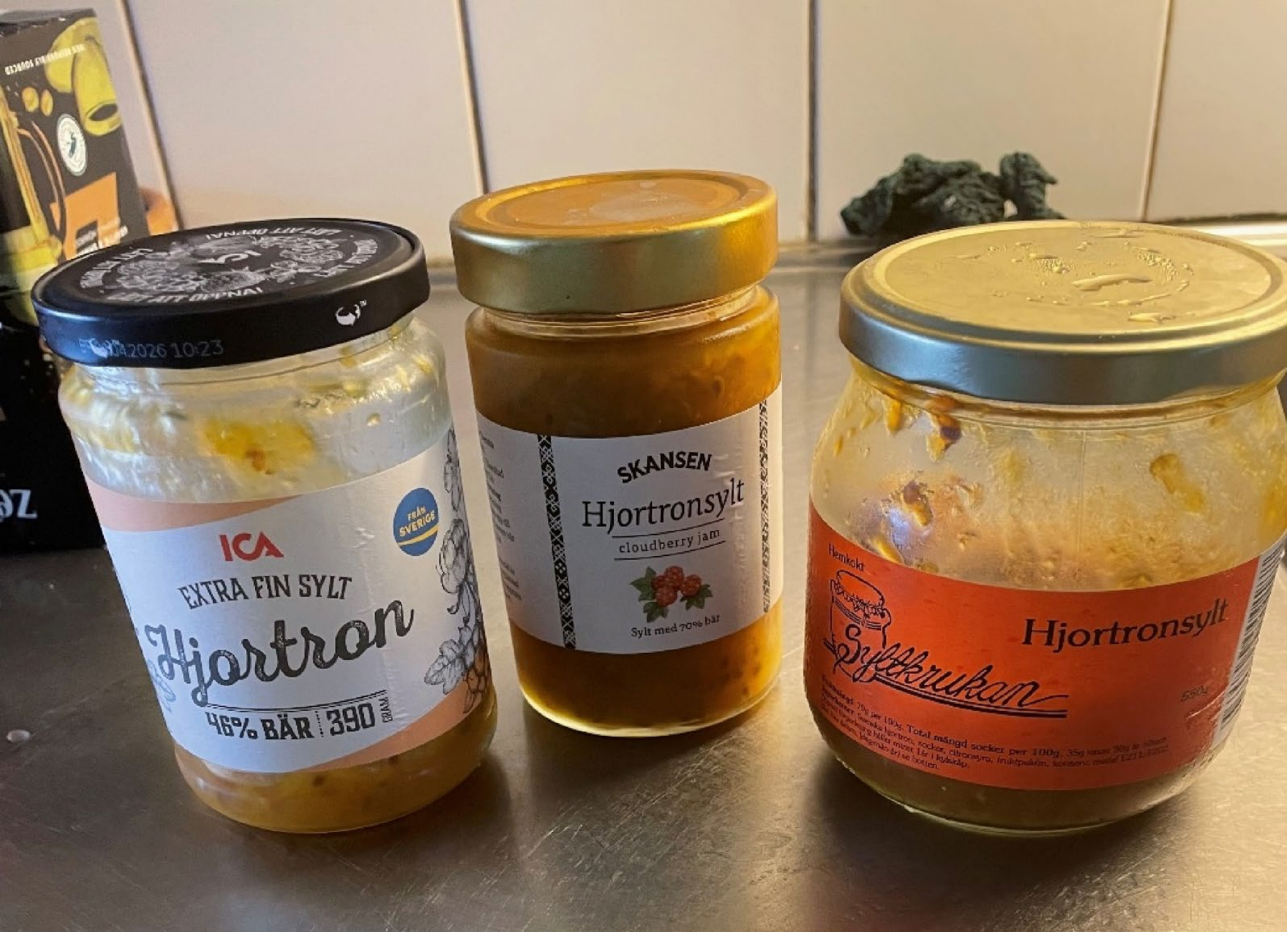
Commercially produced cloudberry jam of various brands (Photo Ingvar Svanberg)

A number of industrial food products are available in supermarkets, such as sour milk with cloudberries (*fjällfil*), cloudberry ice cream, mulled wine (*glögg*), cloudberry sweets, beauty products (facial cream and mask, hand cream, shampoo, etc.) containing cloudberry seed oil, and many others. Cloudberry is used for liqueurs and as flavouring in baked goods. There are many artisanal cloudberry products available especially in the northern part of the country. A local farm shop in the northern town of Piteå sold the following products made of cloudberries in 2025: cloudberry jam, marmalade, candies, tea, sauce for ice cream, and cordial. Figure 14 In recent years, local producers have developed a range of specialised products based on cloudberries, such as cloudberry syrup, which can be served on pancakes or ice cream, or as a sauce with meat and fish. Local food artisans seek to create new products that suit today’s consumers who are curious and constantly looking for new taste experiences. A small enterprise in the northern province of Jämtland has created cloudberry mead prepared with cloudberry juice, birch sap and honey. There is also a gin flavoured with juniper berries and cloudberries sold by a company in the province of Värmland. These exclusive alcoholic beverages are available at Systembolaget, the monopoly company selling alcoholic drinks in Sweden.

### Cultural significance

The cloudberry has taken on a strong cultural significance: it is highlighted as a general symbol of Norrland province and a key ingredient in northern gastronomy [55]. In literature and poetic depictions, cloudberry appears both as a concrete ingredient in stories and as a symbolic representation of the nature and culture of northern Sweden (Norrland, Lapland, Sápmi). The most well-known novel is probably *Hjortronlandet* ‘The Cloudberry Field’ by the famous author Sara Lidman (1923–2004), which was published in 1955 and translated into several languages. Cloudberries are also used as a motif in textile art particularly in northern Sweden. Berries are found as printed motifs on for example children’s clothing (baggy-pants, sweatshirts, etc.). Similarly to adjacent countries Norway and Finland, cloudberries have been depicted on Swedish stamps (1977, 2012). There is also jewellery in the shape of cloudberries, among others tie pins, earrings and necklaces.

Cloudberry stays firmly in the minds of consumers and tourists as a “northern” product since several decades, and the tourism industry in northern Sweden emphasizes the opportunity for visitors to go out into the forests, freely collect cloudberries and take them home, in contrast to neighbouring Norway, where a common policy concerning non-wood forest products allows anyone to gather cloudberries on public property and eat them on location, but only local residents are allowed transport them from the gathering location. In Sweden there are no such restrictions [77].

### Future perspectives

Wild berries for consumption are highly recommended by the Swedish National Food Agency in their dietary guidelines; berries contain vitamins and other important elements for human health and well-being [93] Ecologists also advocate increased use of properly managed game, berries and mushrooms from the forest as sustainable and economically sound food production in the future [5]. Nature’s pantry offers these foods for free; according to experts wild food could significantly contribute to food security, and when properly managed also a better economy in rural areas. Through improving game and forest management, it would be possible to increase the quality of the diet as well. Not only game meat would be part of this process but also wild berries and mushrooms [94]. Swedish forests have for centuries provided humans with a number of important provisioning and cultural ecosystem services. Berries have been gathered for food and trade for more than two hundred years, in some regions for much longer [9, 55, 95]. Hobby foragers like our respondents are deeply convinced that gathering is not a difficult or challenging activity; it encourages a healthier lifestyle and contributes to both physical and mental well-being, as gatherers often remain for many hours outside in nature [96].

Sweden is not the only country harvesting cloudberries; they also grow in Norway and Finland and in other parts of the circumpolar region. In all three countries, cloudberries are nowadays well integrated into the everyday diet. Cloudberries have not yet made their way into global gastronomy, but would have certain prospects if their availability could be secured. The international future of cloudberries remains uncertain, because the fruits have not reached far outside the boreal regions and harvests depend to a high degree on the consequences of climate change.

## Conclusions

Modern Swedish society is urban, information-saturated and globalised: ideas, fashions, foods and goods are quickly brought to and from national and international markets. The physical environment is changing due to climate change: this requires adaptation, and local knowledge and traditions are increasingly substituted by glocal (simultaneously global and local) habits and new fusions. This development is clearly visible in the gastronomy: local or regional foods and ingredients can be brought in from the margins of geography to national and global consciousness within a short time span. They may or may not become part of a globalised foodscape within just a few decades or even years and months. TV and internet are often the means for transmitting culinary news, and chefs focus with gusto nowadays on local and wild dishes or ingredients. The never-ending search for exotic, exciting and special foods and ingredients has moved from regional and national to a global internet-based community consisting of millions of consumers who are hungry for new experiences. Tourist businesses increasingly strive to satisfy this craving for innovative foods and to attract customers who can pay high prices for exotic dishes. When an ingredient, plant or dish becomes mainstream, seasoned and innovation-minded gastrotourists usually move on to something else, leaving the ingredient or dish behind and looking for something more exciting, rare, dangerous, generally unknown or connected to a specific location which is maybe only recently starting to open up to the world. This process happens with increasing velocity also in Sweden.

Cloudberry has made a class journey from a limited northern local food only consumed at the margins of society to an appreciated national and regional Nordic delicacy connected with fine dining and festive occasions. It has the potential to become an exotic global food if promoted by chefs or influencers also outside the Nordic context, because it is both healthy and colourful, challenging to harvest but easy to prepare. Yet, while bilberry and cowberry have reached international markets, cloudberry is still mostly used in the domestic cuisine in Sweden, awaiting its global promotion. The challenges appear to be unsurmountable: small harvests which vary each year, climate change and weather fluctuations, difficult gathering conditions (bogs, mosquitoes, etc.), little success with cultivation and (un)availability of seasonal workers from outside Europe. Producers cannot deliver large or even stable yearly quantities at reasonable prices, even when there have been “cloudberry years” with an abundance of berries. The connection of cloudberry with the far north and specific northern tastes and cuisines, the localisation (in opposite to globalisation) of cloudberry and efforts to define it as a “northern” and national delicacy contribute to keeping the berry to local markets, making it an exclusive Nordic product rather than an international or global delicacy. In any case, enthusiastic hobby gatherers will continue to forage for cloudberry in the bogs and marshlands of Sweden, but the future is uncertain: will cloudberry go back to being just local food, or can the food industry find a way to solve the availability issues and answer to the challenges not only Nature but also humans pose to it?

## Data Availability

No datasets were generated or analysed during the current study.
